# Examinations of the Digestive Organs—Chronic Disease Generally

**Published:** 1913-06

**Authors:** Boardman Reed

**Affiliations:** Los Angeles, Calif


### BUFFALO MEDICAL JOURNAL

Volume 68 JUNE, 1913 No. 11

### ORIGINAL ARTICLES

*No responsibility is assumed by the Journal for the opinions of contributors of original articles; nor for methods of expression nor errors in proof reading, excepting for articles in a foreign language contributed for translation. The right is reserved to decline contributions on account of bona fide lack of space; or because they do not bear on practical medicine and surgery; or because they would fail to interest or would give offense to the majority of subscribers. The expression of minority views or reasonable and courteous criticism of the views of others is not considered offensive.*

### Examinations of the Digestive Organs—Chronic Disease Generally

BY BOARDMAN REED, M.D.
Los Angeles, Calif

THE progress of medical science has been so rapid in these last years that large numbers of physicians are not keeping up with even the discoveries and achievements in the more important practical lines. This is unavoidably true of even the more broadly educated men in the regular profession, especially of those who are kept excessively busy earl yand late with very little time left for study. As for those who have got into practice through any of the side doors after an insufficient course, such as many of the sectarian schools provide, their shortcomings are necessarily great.

In the chronic stomach cases the patients have usually seen various physicians or healers of some sort before consulting a gastrologist, and yet it is rare to meet with a chronic dyspeptic who has been thoroughly examined, even externally, with sufficient care to determine the size and position of the liver, stomach and colon, in addition to the more usual examination of the urine, heart and lungs, yet such an examination is easily made in most cases by a well trained diagnostician with sufficient accuracy to be reasonably decisive, even without the help of the X-ray. The chemical analysis of the stomach contents and stool and microscopical examination of these can sometimes be deferred till later in the less severe cases, since they are not usually practicable, except with the help of a specialist, as is also true of the equally important tests of the motility. But how foolish it manifestly is to put a dyspeptic patient upon a course of any active drugs without at least making the simple external examination which would determine whether or not there is some marked displacement of dilatation of one of the viscera which would absolutely prevent any cure till it had been attended to; and one of the ablest internists has declared that fifty per cent. of all civilized women have enteroptosis.

After getting a full history and account of the patient's symptoms in a stomach case, the first step should be to map out the abdominal organs by palpation (the Pottenger light touch palpation especially) and percussion with a confirmation of the findings in very doubtful cases by means of the X-ray; and in most cases, especially when the complaints include pains in any part of the alimentary canal, or vomiting, constipation or diarrhea, unless they should yield within a few days or weeks at the furthest, there should be a chemical and microscopical examination of the gastric contents and of a stool after specially prescribed meals. These examinations are very necessary to that complete knowledge of the case which should precede any prolonged treatment that might otherwise be on the wrong track and thus do more harm than good. The lack of such thoroughness in reaching a diagnosis accounts for many failures in treatment.

The derangement of the stomach that is one of the most easily cured in an early stage is a simple excess of the hydrochloric acid in the gastric juice—hyperchlorhydria—which is a frequently noted result of too much of the universally used condiments and other stimulating accessories of our food, and, indeed, of merely eating regularly three or four times a day too much food of any kind—and still more of a large excess irregularly, that is at irregular times. And this is the form of indigestion which leading gastrologists now assert to be most commonly found in their patients. It is usually remedied easily at first by regulating the diet and habits of the patient otherwise without even having to prescribe full doses of some mild alkaline drug, which is necessary in advanced cases. When, in such cases, there is a failure to make the diagnosis early, or still worse, when the common mistake is made of diagnosing them vaguely as "stomach trouble" and prescribing hydrochloric acid with or without pepsin, a frequent result after a long continuance of the wrong treatment, is a marked aggravation of the pain and finally either gastric or duodenal ulcer in the site of which cancer not infrequently develops.

This is a trite theme for up-to-date clinicians, but that it needs more discussion for the majority of physicians is only too manifest. The *Journal of the American Medical Association* of March 8th contains a valuable and really important paper which shows nevertheless how even some of our most prominent men fail to appreciate the great usefulness of gastric analysis in settling a doubtful case and enabling the attendant to cure promptly at first a simple functional affection which, neglected for a few months through ignorance of the exact secretory conditions, is likely to become finally a fatal disease. The writer of the paper is one of the distinguished surgeons of this country, Dr. Wm. H. Wathen of Louisville, Ky. He cites several obscure points which have been cleared up by the work of the surgeons through their operations in the abdominal cavity. He says:

"We were told that chronic ulcer in the stomach was usually multiple and ten times more frequent than chronic ulcer in the duodenum. We now know that chronic ulcer in the stomach is multiple in about six per cent. of cases and that we have one ulcer in the stomach to three in the duodenum." These are certainly most important facts which the surgeons have established. He shows also that ulcers of the stomach are more frequent in men than in women, contrary to the prevalent idea, a proportion in fact, of 75 per cent. in men; but he errs in intimating that the same false notion has prevailed until now as to duodenal ulcer. The recent books on diseases of the stomach and intestines—my own certainly and most of the other later works on the subject with which I am familiar, state that duodenal ulcer is more frequent in men. Wathens has evidently been a more careful student of the results of operation on the abdomen than most surgeons, for he admits frankly, in opposition to them, what the internists have always insisted upon, that only a small proportion of gastroinestinal cases need to be turned over to the surgeons. In the same paper he says:

"In 90 per cent. of cases of chronic dyspepsia there is no pathologic lesion in the stomach, and the conditions requiring operation in the entire gastrointestinal tract, and all structures in the abdominal and pelvic cavities, do not exceed 30 per cent." But the statements in Dr. Wathen's paper that will tend to mislead some readers and confirm the indifferent ones in their careless custom of delaying too long in having gastric analysis made are the following:

"The effort to construct a pathology on chemical analysis of the stomach secretions has been a total failure," and again, "while I do not deny that there is value in stomach analysis, it must always be subordinate to the clinical history and the physical examination." Since the surgeons have so clearly demonstrated to us by their operations that in a large proportion of cases gastric cancer forms in the site of an ulcer which, in the beginning at least, is always associated with an excessive secretion of hydrochloric acid, the importance of making a chemical analysis soon after there is a suspicion of an excessive secretion, should surely not be minimized, no matter what expense of time, trouble and money it may involve. And let it be repeated that of all the manifold gastrointestinal troubles, functional and organic, hyperchlorhydria, even though it be a symptom only, is the most prevalent according to a number of the authorities.

While this article is being written, there comes to hand the *"Archiv fuer Verdauungskrankheiten"* of February, 1913, containing a very elaborate paper entitled "A Contribution to the Clinical Diagnosis Between Ulcer of the Stomach and Ulcer of the Duodenum," by Dr. Sommerfeld of Posen, the opening sentence of which is as follows: "Through the works of English and American surgeons especially it has been shown in the last years that ulcer of the duodenum is more frequent than we have hitherto supposed." The writer of the paper cites several Continental authors who have questioned the propriety of following Mayo in accepting as the correct boundary line between ulcers of the stomach and duodenum the vein which crosses the pylorus, because of the variability of its course. Sommerfeld divides into three groups the 44 ulcer cases which he has studied carefully in the German Alexander Hospital in St. Petersburg and determined the localization of them by either operation or section. In 11 of these the ulcer was above the pylorus, 10 of them in the lesser and one in the greater curvature. In 12 the ulcer was within the pylorus, and in 21 it was in the duodenum and mostly within a few centimeters of the pylorus.

In constipation the physician, before advising a dependence upon purgatives or enemata, which is about the worst he could do, should first be able to exclude such surgical affections as tumors and adhesions as well as dilatation or displacement in any part of the digestive tube, and also hyperchorhydria, which, besides its other serious tendencies, markedly conduces to constipation. Dr. Benedict's effervescent test of the stomach contents will often suffice in such a case, without the need of introducing the stomach tube, though a thorough examination of the stomach contents furnishes much more information. In the constipation of babies and in all very stubborn cases pyloric stenosis should be looked for, and in women one should think of a possible retroversion or flexion.

Not only in the manifest disorders of the digestive tract but also in that extremely common group of circulatory diseases, hypertension, arteriosclerosis and Bright's, which so frequently develop insidiously in chronic indigestion, the same thorough examinations of the abdominal viscera followed by a careful regulation of the diet are necessary, if we would succeed in even staying their progress and prolonging life. Excessive indulgence at the table and all kinds of imprudence in eating and drinking, though not necessarily of alcoholic liquors, will generally be found to be prominent among the causes; and to tell the patient that he has been eating too much as well as often too richly, and insist upon a change in his perverted habits in these respects, requires exceptional courage and force in the attendant.

Besides great attention to the diet, which is of chief importance in these affections, various mechanical methods, hydriatic, electric, etc., including also vibration, massage and a sufficient amount of exercise in some form, active or passive according to the nature and stage of the malady, are very valuable adjuvants. Drugs play only a subordinate role here, except that when the heart has begun to fail some cardiac tonic becomes indispensable, and digitalin, Merck's Germanic, has proved in most cases the most satisfactory one.

The proper treatment of virtually all the chronic diseases is likely to require that the family physician, when he retains charge of the patient, should hold consultations with specialists, and often in more than one line, as well as make or have made numerous tedious laboratory examinations. And one who is no longer seeking practice may be permitted to say that not all of our general practitioners are obtaining as much assistance from consultants as their well-to-do patients would be only too glad to pay for in serious cases, or in cases which are entering upon a downward course which tends to end seriously unless they are soon put upon the right track. It is only after extraordinary effort and care, entailing often a very heavy expense, have been employed unavailingly, that scientific medicine can in any case be said to have failed.

---

Angulation of the Junction of the Hepatic and Common Ducts After Cholecystomy. Simulating Common Duct Obstruction. DeWitt Stetten, M. D. *Annals of Surgery,* Feb., 1913.—The author reports a case in New York in which the tension necessary to bring the gall bladder up to the peritoneum for attachment was the cause of the angulation, and intimates that this fault is responsible for failure of a biliary fistula to close where overlooked gallstones or similar obstructions can be ruled out. He recommends more frequent excision of the gall bladder or at least no fixation of the gall bladder which results in tension upward.

